# Effect of clozapine on ketamine-induced deficits in attentional set shift task in mice

**DOI:** 10.1007/s00213-017-4613-x

**Published:** 2017-04-12

**Authors:** M. Szlachta, P. Pabian, M. Kuśmider, J. Solich, M. Kolasa, D. Żurawek, M. Dziedzicka-Wasylewska, A. Faron-Górecka

**Affiliations:** Department of Pharmacology, Institute of Pharmacology Polish Academy of Sciences, Smętna Street 12, 31-343 Kraków, Poland

**Keywords:** Cognitive deficit, Schizophrenia, Set-shifting, Clozapine, Ketamine, Extra-dimensional

## Abstract

**Rationale:**

Clozapine (CLZ) is an effective treatment for schizophrenia, producing improvements in both negative symptoms and cognitive impairments. Cognitive impairments can be modelled in animals by ketamine (KET) and assessed using the attentional set-shift task (ASST).

**Objective:**

Our first aim was to determine whether CLZ improves cognitive function and reverses KET-induced cognitive impairments using the ASST. Our second aim was to assess dose dependency of these effects.

**Results:**

Our findings demonstrate that acute as well as sub-chronic administration of KET cause cognitive deficits observed as increase in number of trails and errors to reach the criterion in the EDS phase. CLZ 0.3 mg/kg reversed the effects of both acute and sub-chronic KET, with no effects on locomotor activity. However, clozapine’s effect after sub-chronic administration of dose 0.3 mg/kg was not as explicit as in the case of acute treatment. Moreover, administration of 1 mg/kg CLZ to KET-treated mice induced or enhanced deficits in the extra-dimensional shift phase compared to 1 mg/kg CLZ administration to mice not receiving KET. Locomotor activity test showed sedation effects of CLZ 1 mg/kg after acute treatment; therefore, effect of CLZ 1 mg/kg on KET-induced cognitive deficits was not evaluated in the attentional set-shift task (ASST) test.

**Conclusions:**

The present findings support dose-dependent effects of CLZ to reverse KET-induced cognitive deficits. The observed dose dependency may be mediated by activation of different receptors, including monomers and/or heterodimers.

## Introduction

Studying the mechanisms of action for antipsychotic drugs requires development of new rodent models. However, modelling symptoms of schizophrenia in animals is complicated by the inability of current approaches to mimic all aspects of such a complex and uniquely human disorder. Most current animal models produce behavioural changes that resemble the positive symptoms of schizophrenia, which probably reflect altered mesolimbic dopamine function. Some models also include altered social interaction, as well as learning and memory impairments, which are analogous to negative and cognitive symptoms of schizophrenia, respectively (Jones et al. 2011; Neill et al. 2014). Prefrontal cortex (PFC) dysfunction is a major contributor to the symptoms of schizophrenia, and includes impaired ability to shift perceptual attentional set. Noncompetitive *N*-methyl-d-aspartate receptor (NMDAR) antagonists such ketamine (KET) and phencyclidine (PCP) produce behavioural effects in healthy people that resemble symptoms of schizophrenia in patients (Krystal et al. 1994; Lahti and Tamminga 1999). Moreover, acute administration of KET or PCP rapidly increases PFC metabolic activity in parallel with PFC-related psychotic symptoms in healthy individuals (Vollenweider et al. 1997). This observation was adapted to an animal model of schizophrenia; acute and/or sub-chronic treatment with KET or PCP, followed by a washout period, induces cognitive impairments in animals (Scheggia et al. 2014; Neill et al. 2010; Becker et al. 2003; Becker and Grecksch 2004; McLean et al. 2008; Kos et al. 2011; Nikiforuk et al. 2013). Cognitive disturbances associated with schizophrenia and depression are closely related to dysfunctions of the prefrontal cortex, a brain region thought to execute cognitive control over behaviour (Shallice 1988). Attentional set shift tasks are used to assess frontal lobe damage and cognitive flexibility. This kind of tasks is based on the use of compound stimuli (i.e. texture and odour stimuli in different modalities). Subjects are trained to pay attention to one dimension on the basis of positive feedback or reinforcement and to ignore the other one, including assessing of reversal (previously correct exemplar of the same dimension is now incorrect and previously incorrect exemplar is now correct), intra-dimensional shifting (novel exemplars are introduced, but the relevant dimension is still the same) and an extra-dimensional shift (novel exemplars are again introduced, but now the previous dimension is irrelevant) (Keeler and Robbins 2011). In humans, the classical tests to measure cognitive abilities are the Wisconsin Card Sorting Test (WCST) (Grant and Berg 1948; Berg 1948; Eling et al. 2008) and the CANTAB ID/ED task (Roberts et al. 1988; Dias et al. 1996), which are examples of attentional set shift tasks. The WCST is a test of card sorting in which presented cards to patients differ in the colour, the shape and the numbers and patients have to match cards not knowing the rules. Following decision about matching stimulus cards is based on feedback whether a particular match to an exemplar is right or wrong. In the CANTAB IED, test stimuli (different sets of shapes overlaid by lines) are presented on a touch-sensitive screen where only one of the shapes is initially relevant. The CANTAB test involves main forms of cognitive flexibility—reversal learning and extra-dimensional shift; however, WCST lets to assess extra-dimensional (ED) shifting. Birrell and Brown (2000) adopted ID/ED test for rodents—rats (Birrell and Brown 2000) and mice (Bissonette et al. 2008)—using different textures and odours as perceptual dimension. Overall, the attentional set shift task version for rodents is now one of the main animal tests to evaluate cognitive deficits (Barnett et al. 2010).

The first aim of our study was to determine whether the antipsychotic, clozapine (CLZ), reverses KET-induced deficits, using the ASST model. CLZ is the most effective drug for treatment-resistant schizophrenia (Elkis and Buckley 2016), and is preferred because it lacks extrapyramidal side effects (EPSE) and produces sustained prolactin elevation and improvements in negative symptoms. CLZ also has a unique antisuicidal effect. There is extensive literature regarding the beneficial effects of CLZ for reducing cognitive deficits in patients with schizophrenia (Hill et al. 2010). Spagna et al. (2015) demonstrated that treatment with CLZ significantly improves orienting, but does not alter executive control of attention. Furthermore, CLZ has a unique mechanism of action compared to other antipsychotic drugs (Leo and Regno 2000). Most antipsychotics produce 70% or greater dopamine receptor 2 (D2) occupancy at clinical doses; however, CLZ produces optimal antipsychotic efficacy with 50–60% D2 occupancy. CLZ reaches this receptor occupancy with 300–400 ng/mL doses, and low-dose CLZ also produces complete occupancy of serotonin 5-HT2 receptors. Moreover, CLZ has high affinity for D4 receptors and is an antagonist at adrenergic, cholinergic (muscarinic, M1 and M5), histaminergic, and serotonergic receptors (Baviera et al. 2008). The antipsychotic effects of CLZ are associated with rapid dissociation from D2 receptors, preferential action at serotoninergic 5-HT2A and 2C receptors, 5-HT1A receptor agonism, and increased extracellular levels of acetylcholine in the PFC, striatum, and nucleus accumbens (Horacek et al. 2006). Moreover, low D2 occupancy may explain the absence of EPSE and sustained prolactin elevation. Although CLZ is a very effective antipsychotic, it is usually a last treatment option in patients with schizophrenia, due to the risk of agranulocytosis. Other side effects of CLZ include skin rashes and effects on the cardiovascular, nervous, and digestive systems. However, numerous studies have demonstrated the efficacy of CLZ, including reversal of the effects of KET on a social interaction test (Becker and Grecksch 2004). CLZ blocks the psychotomimetic effects of KET in patients with schizophrenia (Malhotra et al. 1997). We used a modified ASST to assess PFC-mediated cognitive flexibility in mice (Birrell and Brown 2000; Papaleo et al. 2008; Scheggia et al. 2014; Kos et al. 2011). Previous research has administered acute or sub-chronic NMDAR antagonists; therefore, we administered similar acute injections of KET (Kos et al. 2011), as well as applying sub-chronic administration (McLean et al., 2008). The detailed information can be provided in the materials and methods section.

The second aim of our study was to assess whether the effects of CLZ on ASST performance are dose dependent. In our previous studies using an in vitro model, we demonstrated the effects of CLZ on D1-D2 receptor heterodimers. The effect was strongly CLZ concentration dependent; lower concentrations, which result in binding to high affinity sites, decreased the physical interaction between these two dopamine sub-receptors, whereas higher concentrations of CLZ increased physical interactions (Faron-Górecka et al. 2008). Concentration-dependent actions of CLZ were also observed in studies of 5HT1A-D2 heterodimers (Lukasiewicz et al. 2011). Therefore, we studied two doses of CLZ, and assessed ASST performance in mice.

Our data may elucidate the complete mechanisms of action of CLZ and therefore contribute to eliminating the limiting side effects of this clinical option for the treatment-resistant symptoms of schizophrenia.

## Materials and methods

### Animals

Experiments were conducted on male C57Bl/6J mice (approximately 26 g and 11 weeks of age; Charles-River, Germany). Mice were housed in standard laboratory cages under standard colony conditions (21 ± 2 °C, humidity 40–50%) and on a 12-h light/dark cycle (lights on at 07:30). Mice were housed in five per cage with mild food deprivation (2.9 g of food pellets per day) and ad libitum access to water for 2 weeks before the test. Food deprivation was crucial because mice at 85% of their initial body weight would eagerly feed from containers to obtain the food reward. Breeding five mice per cage was our limitations; however, according to our experience, suppling five pieces of approx. 2.9 g food pellet into one cage leads to appropriate reduction of mice weight within 14 days i.e. weight range for the mice at the beginning of food deprivations was 24.5–27.5 g and for the end of the 14-day-period of food restriction the weight range was 20.0–23.5 g. The experiments were approved by the Ethics Committee for Animal Experiments, Institute of Pharmacology, Poland.

### Chemicals

KET (10% aqueous solution of 115.34 mg/mL, Biowet, Poland) was dissolved in saline (SAL) to obtain a concentration of 100 mg/kg. CLZ (Tocris, UK) was dissolved in 1 M hydrochloric acid and then diluted in SAL. NaOH solution was added to buffer the solution to ∼pH 6.5–7.0. Experiments were conducted using two different paradigms: acute and sub-chronic administration. For the acute paradigm, drugs (SAL, KET, and/or CLZ at 0.3 mg/kg; intraperitoneally, i.p.) were administrated 1 h before start of the ASST, on each test day. For the sub-chronic paradigm, drugs (KET or SAL) were repeatedly administrated (i.p.) for 7 consecutive days, followed by replacement with CLZ (0.3 or 1 mg/kg; i.p.) for the next 7 days. All injections were done once per day during this period. The ASST was performed following 14 days of treatment.

### General rules of the ASST

The ASST was conducted as described by Kos et al. (2011). The apparatus was constructed from black plywood and had a wire grid floor. It consisted of a larger waiting compartment and two equally sized choice compartments, which were accessed through sliding doors. There was a bowl of water in the waiting compartment, whereas there were containers with food rewards covered by a layer of digging medium in the choice compartments. The containers with food rewards differed from each other based on the digging media and odours on the outer wall of the containers (Table 1).

**Table 1:** Types of digging media and odours used in the attentional set-shift task (ASST)

	Set 1	Set 2	Set 3	Set 4
Digging medium	Fine gravel vs beads	Corkboard vs aluminium balls	Cardboard vs plastic	Bamboo sticks vs polystyrene
Odour	Grapefruit vs pine	Green tea vs coconut	Clove vs cherry	Peach vs rosemary

### ASST procedure

The ASST procedure consisted of 7 days of adaptation to the new place, 14 days of food restriction with handling, habituation performed during 1 day, and 2 days of test (Heisler et al. 2015; Kos et al. 2011). Habituation typically occurred 1 or 2 days before testing, and testing occurred on two consecutive days. Habituation involved five phases of adaptation. During the first phase, mice were habituated to the testing area by spending 5 min in the waiting compartment. In the next four phases, mice were trained to dig in pots filled with sawdust, in order to retrieve a food reward. Mice started each of the four phases in the waiting compartment, and the sliding door was then raised to allow entry to the test compartments containing the pots with food rewards. In the second phase, mice ate food rewards placed at the bottom of empty containers. During the third phase, food rewards were placed on top of a thin layer of sawdust, and in the fourth phase the rewards were covered by a thin layer of sawdust. During the fifth phase, the reward was covered by a thicker layer of sawdust (∼2 cm). All phases, except for phase 5, consisted of two trials of the mouse eating a food reward from each of the two containers. The fifth phase included three trials.

The first test day began with adaptation to the apparatus and a brief reminder (repetition of the fifth phase of habituation), followed by the five different phases of the task: (Avery and Krichmar 2015) simple discrimination (SD), (Barnett et al. 2010) compound discrimination (CD), (Baviera et al. 2008) compound discrimination reversal (CDR), (Becker and Grecksch 2004) intra-dimensional shift (IDS), and (Becker et al. 2003) intra-dimensional shift reversal (IDSR). Each phase consisted of three training free trials and several test trials, which varied depending on whether mice chose the correct container. During training, the door remained open even if the choice was incorrect, and the mouse may then have investigated and collected the food reward from the opposite container. Free trails were not scored, and the results from these free trails were not taken into consideration in the presented results. However, during the test, incorrect choices were recorded as errors, the door was closed, and the trial was terminated. In order to complete each phase, mice had to reach a criterion of eight correct discriminations out of ten consecutive trials. Wrong discrimination was considered as digging in medium—just having a look was not recognize as mistake. The second test day consisted of four different phases: (Avery and Krichmar 2015) intra-dimensional shift 2 (IDS2), (Barnett et al. 2010) intra-dimensional shift reversal 2 (IDSR2), (Baviera et al. 2008) extra-dimensional shift (EDS), and (Becker and Grecksch 2004) extra-dimensional shift reversal (EDSR). The phases for both test days are described in Table 2. At the beginning of each discrimination, dust of food reward (chocolate balls) was sprinkled over all pots to avoid a scent cue from the food reward. The order of exemplar medium-odour pairings was counterbalanced; the equal number of mice began the test from set 1, set 2, set 3, set 4 of medium-odour pairing.

**Table 2:** Description of attentional set-shift task (ASST) phases

First test day	
SD	Discrimination on 1 dimension only—digging media; 1 medium is correct, e.g. fine gravel
CD	Correct digging medium is the same as for SD, e.g. fine gravel; additional dimension, odour, is introduced but is irrelevant (e.g. grapefruit and pine)
CDR	Correct digging medium is the other medium from the same pair of media, e.g. beads; odour is still irrelevant
IDS	Relevant dimension is still the digging medium and odour is irrelevant; the correct medium is the same as for the next set of digging medium and odour, e.g. corkboard
IDSR	Correct digging medium is the other medium from the same pair of media, e.g. aluminium balls; odour is still irrelevant
Second test day	
IDS2	Relevant dimension is still the digging medium and odour is irrelevant; the correct medium is the same as for the next set of digging medium and odour, e.g. cardboard
IDSR2	Correct digging medium is the other medium from the same pair of media, e.g. plastic; odour is still irrelevant
EDS	Relevant dimension is odour and digging medium is irrelevant; correct scent is a scent from an odour pair derived from a set of 4, e.g. peach
EDSR	Correct scent is the other odour from the same pair of odours, e.g. rosemary. Digging medium is irrelevant

Direction of shift was not counterbalanced, and shift from medium to odour is the only shift direction used

*SD* simple discrimination, *CD* compound discrimination, *CDR* compound discrimination reversal, *IDS* intra-dimensional shift, *IDSR* intra-dimensional shift reversal, *IDS2* intra-dimensional shift, day 2, *IDSR2* intra-dimensional shift reversal, day 2, *EDS* extra-dimensional shift, *EDSR* extra-dimensional shift reversal

### Locomotor activity test

Locomotor activity was measured individually for each mouse using OPTO-M3 locomotor activity cages (Columbus Instruments, Columbus, OH, USA) attached to a compatible personal computer. Each cage (13 × 23 × 15 cm) was connected to an array of photocell beams. Each photobeam interruption was recorded as horizontal activity, which comprised the ambulation scores. The experimental design for locomotor activity measurements was the same as described for the ASST; however, different animals completed the locomotor activity test and the ASST.

### Data analyses

The number of trials to reach criterion in each phase was calculated for each mouse, as well as the number of errors in each phase and duration of time required to complete each phase. Presented results do not include free trials; however, analysing data, taking into consideration this training trial, shows an ID/ED difference in the control group. Statistical analyses for all behavioural tests were performed using two-way ANOVA with post hoc multiple comparison tests (GraphPad Prism 7.0, San Diego, CA).

## Results

### Locomotor activity

**Fig. 1:**
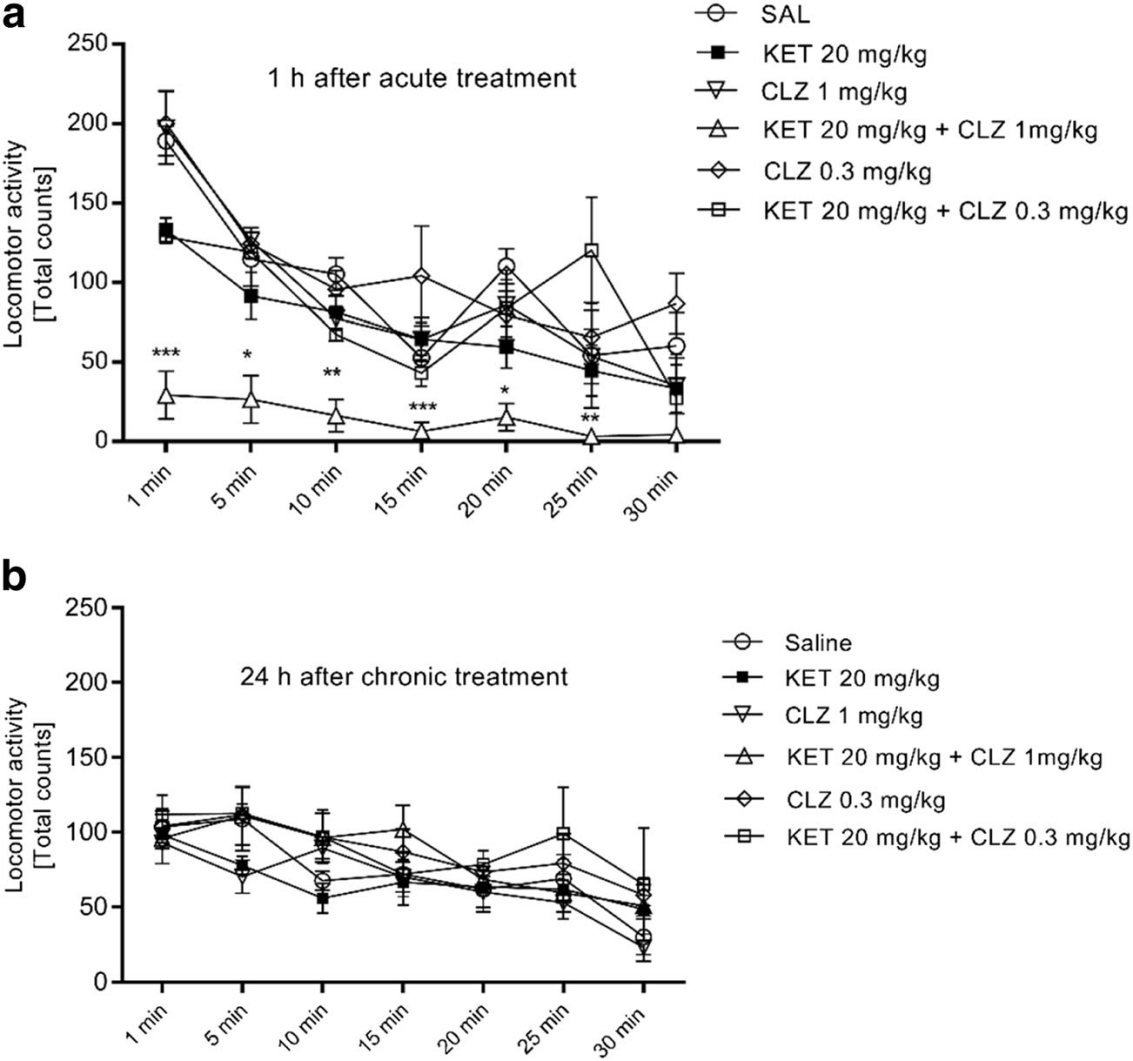
**a** The effect of acute (1 h before locomotor activity testing) treatment on locomotor activity in mice. Data presented as mean ± S.E.M. activity counts over a 30-min test in 5-min periods. Significant changes were observed in the KET 20 mg/kg + CLZ 1 mg/kg group vs the control group (SAL), *p < 0.05, **p < 0.01, ***p < 0.0001. **b** The effect of sub-chronic treatment on locomotor activity in mice. Behavioural test of Locomotor Activity was made 24 h after last treatment. Data presented as mean ± S.E.M. activity counts over a 30-min test in 5-min periods. No significant difference between the groups was observed

Analyses of locomotor activity after acute treatment revealed a significant interaction between time (5 min intervals for a total of 30 min) and drug treatment (SAL, KET, CLZ 0.3 mg/kg, KET+CLZ 0.3 mg/kg, CLZ 1 mg/kg, KET+CLZ 1 mg/kg) [*F*_(30,168)_] = 2.09, *p* < 0.01. There were also significant main effects of time [*F*_(6,168)_] = 29.11, *p* < 0.0001 and drug treatment [*F*_(5,168)_] = 30.35, *p* < 0.0001. Multiple comparison tests indicated that there was a significant decrease in locomotor activity at all time points for the KET+CLZ 1 mg/kg group (Fig. 1a). Two-way ANOVA of the sub-chronic treatment data found no interaction between time and drug treatment [*F*_(30,168)_] = 0.58, ns, although significant main effects occurred for time [*F*_(6,168)_] = 10.64, *p* < 0.0001 and drug treatment [*F*_(5,168)_] = 3.84, *p* < 0.01. However, post hoc analyses did not indicate any significant differences between individual groups. Furthermore, we did not observe significant differences between the SAL and KET groups in either experiment (Fig. 1b).

### ASST performance

**Fig. 2:**
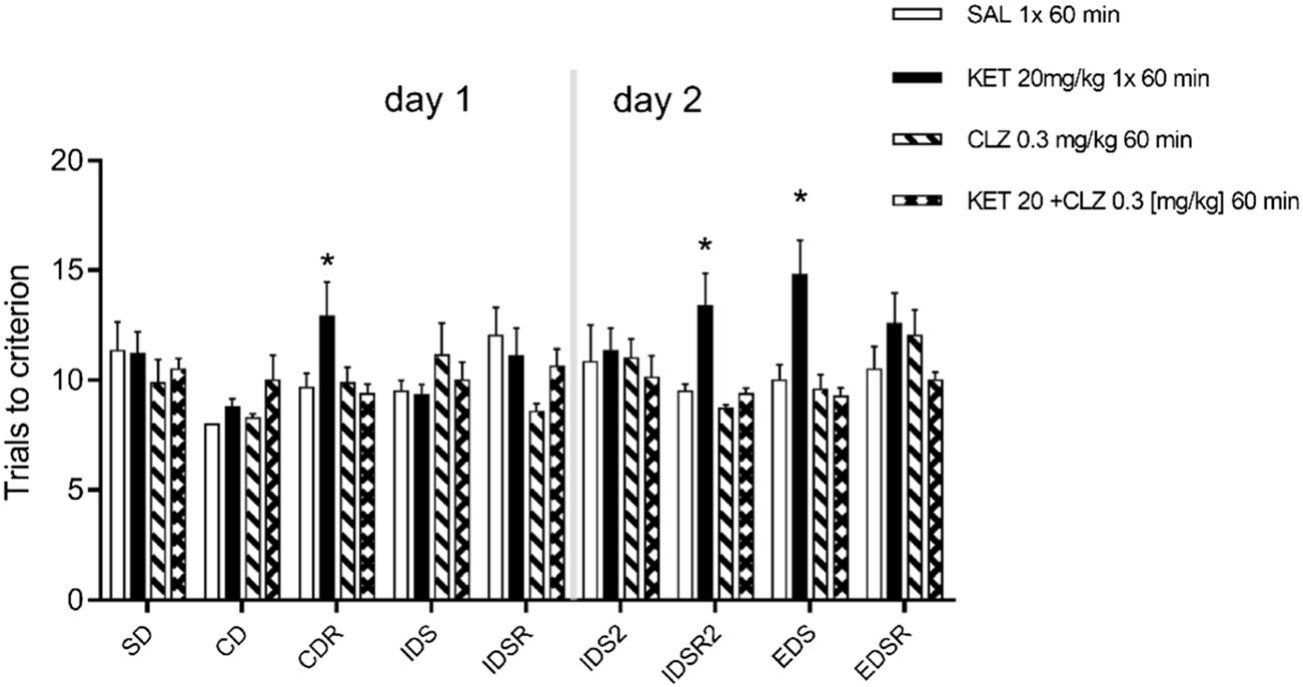
The effect of acute (1 h before ASST) treatment of KET (20 mg/kg) and CLZ (0.3 mg/kg) on trials to reach criterion in attentional set shift task. Data are presented as mean ± S.E.M and were analysed using two-way ANOVA and post hoc multi comparison test. *p < 0.05 compared KET 20 mg/kg vs all groups in CDR, IDSR2, and EDS phase

In total, 80 mice were successfully trained and tested. Mice that did not learn the ASST within 25 trials were not included in the statistical analyses. The treatment groups for the acute experiment were the following: SAL (*n* = 7), KET (*n* = 9), CLZ 0.3 mg/kg (*n* = 7), and KET+CLZ 0.3 mg/kg (*n* = 8). For the sub-chronic experiment, the following groups were examined: SAL+SAL (*n* = 8), KET+SAL (*n* = 9), CLZ 0.3 mg/kg (*n* = 8), KET+CLZ 0.3 mg/kg (*n* = 7), CLZ 1 mg/kg (*n* = 8), and KET+CLZ 1 mg/kg (*n* = 9). Drug treatment did not have any effect on performance during habituation or training. For acute treatment experiments, two-way ANOVA (drug treatment: SAL, KET, CLZ 0.3 mg/kg, KET+CLZ 0.3 mg/kg × set shifting phase: SD, CD, CDR, IDS, IDSR, IDS2, IDSR2, EDS, EDSR) demonstrated a significant interaction [*F*_(24,234)_] = 1.701, *p* < 0.05, as well as significant main effects of the set-shifting phase [*F*_(8,234)_] = 2.046, *p* < 0.05, and drug treatment [*F*_(3,234)_] = 7.662, *p* = 0.0001. Post hoc multiple comparison tests revealed significant differences in a crucial phase of cognitive impairment, EDS, between KET and KET+CLZ 0.3 mg/kg treatments. KET administration significantly increased the number of trials to criterion compared to SAL and CLZ 0.3 mg/kg (from 10 ± 0.73 for SAL and 9.43 ± 0.43 for CLZ to 14.78 ± 1.60 for KET). This deficit was significantly attenuated by combined treatment with KET+CLZ 0.3 mg/kg (9.57 ± 0.68, *p* < 0.05) (Fig. 2).

**Fig. 3:**
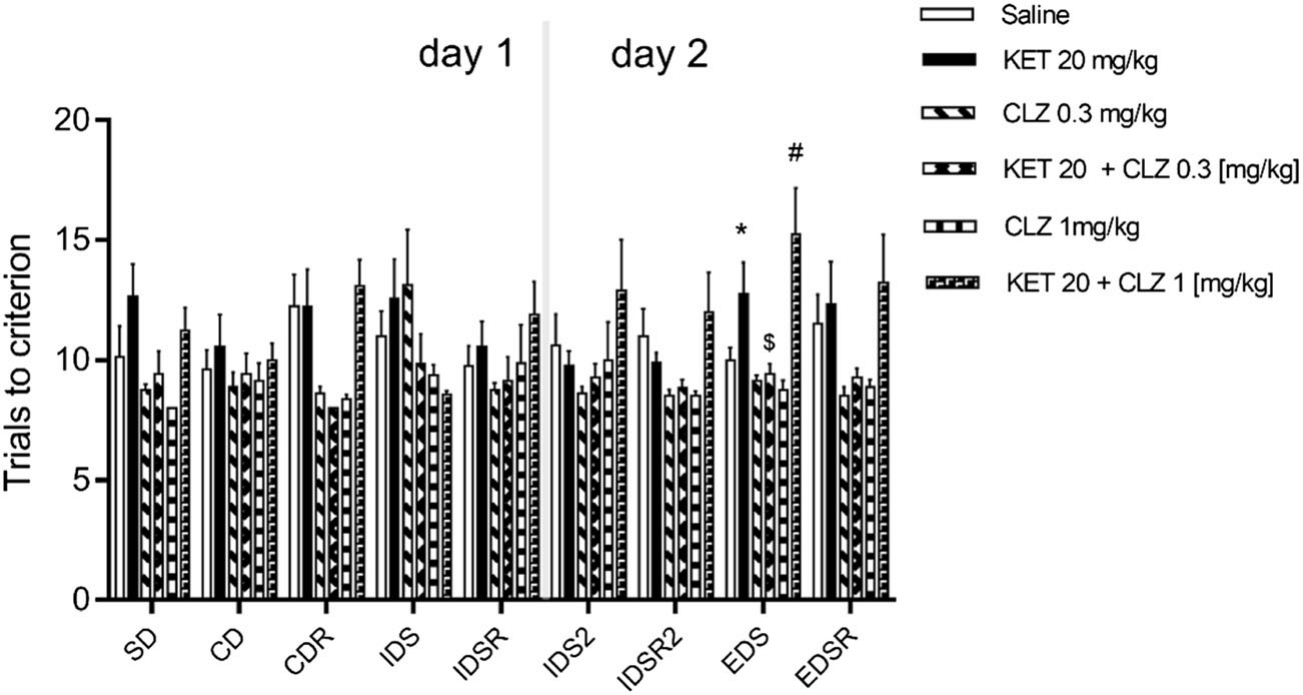
The effect of sub-chronic treatment of KET (20 mg/kg) and CLZ (0.3 or 1 mg/kg) on trials to reach criterion in attentional set shift task. Data are presented as mean ± S.E.M and were analysed using two-way ANOVA and post hoc multicomparison test. *p < 0.05 compared KET vs SAL, #*p* < 0.01 compared KET+CLZ 1 mg/kg vs CLZ 1 mg/kg and $*p* < 0.01 compared KET+CLZ 0.3 mg/kg vs KET in the EDS phase

We also observed significant changes in the EDS phase following sub-chronic KET, although these changes were reduced compared to those following a single administration of KET (from 10 ± 0.5 to 12.78 ± 1.29, *p* < 0.05; *t* test). Two-way ANOVA revealed no significant interaction between drug treatment (SAL, KET, CLZ 0.3 mg/kg, KET+CLZ 0.3 mg/kg, CLZ 1 mg/kg, KET+CLZ 1 mg/kg) and set-shifting phases (SD, CD, CDR, IDS, IDSR, IDS2, IDSR2, EDS, EDSR) [*F*_(40,387)_] = 1.299, *p* = 0.112. There was also no main effect of the set-shifting phase [*F*_(8,387)_] = 0.921, *p* = 0.499. Two-way ANOVA demonstrated a significant main effect of drug treatment [*F*_(5,387)_] = 12.54, *p* < 0.0001. Administration of 1 mg/kg CLZ to KET-treated mice induced deficits in the EDS phase (*p* < 0.01) compared to 1 mg/kg CLZ administration in the absence of KET (trials to criterion CLZ 1 mg/kg = 8.75 ± 0.42, KET+CLZ 1 mg/kg = 15.22 ± 1.96), whereas the lower dose of CLZ (0.3 mg/kg) significantly decreased EDS phase deficits in KET-treated mice (from 12.78 ± 1.29 to 9.43 ± 0.42, *p* < 0.01) (Fig. 3).

## Discussion

The main aim of the present study was to assess whether CLZ reduces KET-induced deficits, using the ASST in a mouse model. Since both NMDAR antagonists and CLZ affect locomotor activity, we first examined the effects of KET and CLZ using a locomotor activity test. Studies assessing locomotor activity and novel object recognition (NOR) following administration of NMDAR antagonists indicate that 5-HT2AR antagonism, 5-HT1AR agonism, and 5-HT6R and 5-HT7R antagonism contribute to attenuating NMDAR antagonist-induced increases in locomotor activity, and also reverse impairments in NOR after acute or long-term treatment with NMDAR antagonists (Meltzer et al. 2011; McOmish et al. 2012). The present study found decreased locomotor activity with the interaction of KET and CLZ at a higher dose (1 mg/kg). CLZ has high affinity for 5HT2ARs; therefore, the observed effect may result from CLZ and KET activity at this receptor. In studies using the NMDAR antagonist, MK-801, CLZ was administered at a low dose (0.2 mg/kg; subcutaneous, s.c.), because higher doses produced disturbances in locomotor activity (Hoffman and Basurto 2014). We did not observe any statistically significant changes in locomotor activity after acute administration of KET (20 mg/kg) combined with CLZ (0.3.mg/kg). However, since locomotor effects were observed after administration of acute KET 20 mg/kg + CLZ 1 mg/kg, locomotor activity was assessed 24 h after the last dose of drugs in the sub-chronic administration paradigm; we did not observe any changes in locomotor activity. The design of our ASST experiments was based on this observation. The higher dose of CLZ (1 mg/kg) was eliminated after acute administration, since complete blockade of locomotor activity (1 h after drug administration) impaired the ability to complete the ASST experimental paradigm. Mice should be fully functioning while completing the tasks, and deficits in cognitive symptoms induced by KET are observed after 50 min of KET treatment (Kos et al. 2011). Our ASST results demonstrate that a single administration of KET caused disturbances in the EDS phase, which reflects cognitive disruption. The dose of KET (20 mg/kg) was selected based on Kos et al. 2011, who found that this dose produces maximal effects in the EDS phase.

In the present study, mice were unable to perform all tests, including SD, CD, CDR, IDS, IDSR, EDS, and EDSR in 1 day; therefore, the ASST occurred over 2 days. In addition, our experimental paradigm included two other sessions, IDS2 and IDSR2, similarly to the procedure described by Kos et al. (2011). These authors found that the additional phases enhance a set of cognitive deficits and produce overall improvements from CD to IDSR2, in addition to deterioration during the crucial phase for cognitive impairment, EDS (Garner et al. 2006; Kos et al. 2011).

Our results indicate that saline-treated mice required similar number of trials to reach criterion to complete the IDS and EDS phases, which might indicate that an attentional set had not been formed. We decided to present results without including free trials because according to literature, authors of many papers did not score the free trials and show ASST results which do not include training trials (Nikiforuk et al. 2015; Heisler et al. 2015; McLean et al. 2008). However, at the beginning, we analysed ASST results taking into account free trials and we observed an ID/ED difference in the control group [mean ± S.E.M. respectively: 12 ± 0.5 vs 13 ± 0.73]. Based on these results, we regarded the ASST procedure as implemented well, and although results without including free trials did not show that ED reflects a ‘cost’ of shifting attentional set from one dimension to another in the control group, we recognized that we can interpret effects of various ketamine/clozapine dosing regimens on ED performance as an effect on attentional set-shifting. In these free trials, mice were allowed to explore both containers regardless of whether they find food reward or not, and this was time for them to learn what cue is relevant. This may explain lack of significant EDS and reversal impairments in the control group because these mice did not have any cognitive deficits and free trails were sufficient to shift their attentional set. It is possible that saline-treated mice learned the EDS rule within these three free trials; thus, they did not need more trails to reach the criterion; this may also explain the occurrence of minimum trials to criterion without any apparent errors in certain discriminations. The same issue has been discussed by McLean et al. (2008). To eliminate the possibility that mouse follows the smell of the hidden food pellets, we sprinkled dust of food pellet over all pots few times during each stage; therefore, we were sure that we avoid scent cue from food reward. Moreover, we took care of a thick layer of medium in containers—in the ASST test, we used glass crystallizers filled to three quarters height by medium which completely prevented getting out of odour. However, regardless of free trails in our ASST procedure, we observed a significant increase in trials required to reach criterion in ketamine-treated mice in the EDS stage, suggesting that acute and sub-chronic ketamine causes a selective deficit in attentional set-shifting ability. Our data confirm the results presented by Kos et al. (2011) demonstrating that, in the mouse model of ASST, KET produced specific deficits in cognitive flexibility after a single dose given 50 min before the test. In our studies, we observed this effect after 60 min of treatment. In the case of chronic treatment, there was data concerning chronic treatment with KET; however, our observations are consistent with results obtained with different NMDA antagonists (PCP) in mice (Scheggia et al. 2014) or chronic KET in rats (Nikiforuk and Popik 2012).

Single or sub-chronic administrations of KET, PCP, or MK-801 are often used in animal models of NMDAR antagonist-induced cognitive disorders. Our data confirm the results presented by Kos et al. (2011) demonstrating that, in the ASST mouse model, KET produces specific deficits in cognitive flexibility after a single dose administered 50 min before the test. The present study demonstrated this effect after 60 min of treatment. The main finding of the present work is that CLZ, at a dose of 0.3 mg/kg, reversed the effect of KET after both acute and sub-chronic treatments. We have therefore provided the first demonstration of the effect of CLZ on KET-induced alterations in an ASST paradigm.

CLZ has previously been tested using set-shifting tasks in rats, rabbits, or mice, in relation to cognitive deficits induced by MK-801 or PCP. CLZ (5 mg/kg) significantly reversed the effects of MK-801 in rats (Jones et al. 2014). CLZ (0.63 mg/kg, s.c.) also reversed PCP-induced cognitive deficits in Morris’ water maze, although the highest dose (1.3 mg/kg) did not produce significant effects (Didriksen et al. 2007). Furthermore, CLZ (0.5 mg/kg) prevented PCP-induced cognitive impairments (Beraki et al. 2008), and at a lower dose (0.2 mg/kg, s.c.) it also prevented MK-801-induced deficits in a NOR test in rabbits (Hoffman and Basurto 2014). These results are consistent with our data indicating that lower doses of CLZ impact the effects of NMDAR antagonists. Our weak effect of KET in the sub-chronic paradigm may have resulted from the length of administration; however, relevant data regarding NMDAR antagonist administration do not enable definitive conclusions. Rat studies have found that KET treatment for 10 days is required to induce long-term cognitive impairments (Nikiforuk and Popik 2012). However, PCP administered to rats twice daily for 7 consecutive days affects the EDS phase of the ASST after a 10-day washout period (McLean et al. 2008). Although Enomoto and Floresco (2009) reported that 5 days of KET administration was insufficient to induce deficits, other research has found that a 5-day treatment with KET induced changes in social behaviour and latent inhibition in rats (Becker et al. 2003). Despite no effects of sub-chronic KET (the effect of acute KET treatment was greater), CLZ 0.3 mg/kg can reverse the effects of KET, whereas a higher dose of CLZ potentiates the effects of KET.

The present findings indicate that the dose of CLZ must be considered in studies of cognitive deficits, as dose dependency occurs, by which CLZ can potentiate (higher dose) or attenuate (lower dose) the effects of KET. In addition, the mechanisms of CLZ-induced changes to KET-induced cognitive deficits have not been elucidated. Connectionist models have demonstrated that the cognitive symptoms of schizophrenia result from dopaminergic neurons altering the gain of activity in working memory (Cohen and Servan-Schreiber 1992; Braver and Cohen 1999). In contrast, instabilities in cortical attractor states can produce the symptoms of schizophrenia (Rolls et al. 2008). Furthermore, increasing or decreasing dopamine neurotransmission using dopaminergic agents potentiates or attenuates KET-induced amnesia in a model of cognition. These data suggest a possible interaction between the NMDA and dopaminergic systems in behavioural and cognitive responses (Farahmandfar et al. 2016). The mechanism underlying these changes may be an imbalance of dopamine D1/D2 receptors, which can alter NMDA and GABA conductance (Avery and Krichmar 2015). We observed a dose-dependent effect of CLZ in KET-treated mice; a lower dose of CLZ reversed the effects of KET, whereas a higher dose enhanced the effects. Such findings may be related to an imbalance of dopamine receptors. Our previous study using an in vitro model demonstrated that a lower dose of CLZ (acting on dopamine receptors in high affinity states, which are the active state of receptors) uncoupled D1-D2 heterodimers, whereas a higher dose of CLZ increased D1-D2 heterodimerization; these scenarios produced different effects on the intracellular signalling pathway involving phospholipase C-mediated calcium mobilization (Faron-Górecka et al. 2008).

The dose-dependent effects of CLZ observed in the present study may also result from action at serotonin 5HT2A receptors, thereby contributing to attenuation of cognitive deficits induced by KET. Previous research has suggested that strong 5HT2A receptor antagonism, combined with weak dopamine D2 receptor antagonism, is the principal pharmacologic feature that differentiates clozapine from other atypical antipsychotic drugs (APDs) and from typical first-generation APDs (Meltzer et al. 2011). This interpretation is supported by data demonstrating that Akt phosphorylation via 5HT2A receptors is required for CLZ efficacy in blocking MK-801 and PCP-induced models of schizophrenic behaviours in mice (Schmidt et al. 2014). These data indicate that CLZ acts as an agonist at 5HT2A receptors. Although higher doses of CLZ produce antagonistic effects at 5HT2A receptors, it is possible that lower doses of CLZ promote Akt phosphorylation via 5HT2A-D2 receptor heterodimers. The potential for these two receptors to dimerize has been demonstrated by Łukasiewicz et al. (2010).

Our study is not without limitations, e.g. the lack of a significant impairment in performance of vehicle-treated animals from the IDS to the EDS phase. The use of three free trials, as previously mentioned, before each new phase of the task is an explanation for this. A similar effect was observed by McLean et al. (2008) in rats. Also, having used only two doses of CLZ is another limitation; a dose response should ideally have been conducted; however, as in our previous in vitro studies, we decided to use only these two doses. A further limitation of the current experimental design was the decision not to counterbalance the dimension shifts, i.e. all the mice were switched from medium to odour. The same issue has been discussed by McLean et al. (2008). Another aspect which could have been a potential weakness was the digging media pairings.

## Conclusion

The present data indicate that CLZ dose-dependently reverses KET-induced cognitive deficits. These behavioural results should be further investigated using biochemical studies, in order to assess whether dose dependence corresponds to mechanisms at different receptors. Specifically, the contributions of monomeric receptor states and heterocomplexes should be considered.
